# Norms and stigma regarding pregnancy decisions during an unintended pregnancy: Development and predictors of scales among young women in the U.S. South

**DOI:** 10.1371/journal.pone.0174210

**Published:** 2017-03-22

**Authors:** Whitney S. Rice, Bulent Turan, Kristi L. Stringer, Anna Helova, Kari White, Kate Cockrill, Janet M. Turan

**Affiliations:** 1 Department of Psychology, College of Arts and Sciences, University of Alabama at Birmingham, Birmingham, AL, United States of America; 2 Department of Medical Sociology, College of Arts and Sciences, University of Alabama at Birmingham, Birmingham, AL, United States of America; 3 Department of Health Care Organization and Policy, School of Public Health, University of Alabama at Birmingham, Birmingham, AL, United States of America; 4 Sea Change Program, Tides Foundation, Oakland, CA, United States of America; Stellenbosch University, SOUTH AFRICA

## Abstract

**Background:**

Norms and stigma regarding pregnancy decisions (parenting, adoption, and abortion) are salient to maternal well-being, particularly for groups disproportionately affected by unintended pregnancy. However, there are few validated measures of individual-level perceptions of norms and stigma around pregnancy decisions. Additionally, little is known about variation in the content of norms regarding pregnancy decisions, and in stigma related to violations of these norms, across socio-demographic groups.

**Methods:**

To create measures of perceived norms and stigma around pregnancy decisions, we developed and pre-tested 97 survey items using a mixed methods approach. The resulting survey was administered to 642 young adult women recruited from health department clinics and a public university campus in Birmingham, Alabama. Principal components factor analyses, reliability analyses, independent t-tests, and correlation analyses were conducted to establish the reliability and validity of scales. Additionally, multiple linear regression was used to identify demographic predictors of higher scale scores.

**Results:**

Factor analyses revealed four subscales for each pregnancy decision: conditional acceptability, anticipated reactions, stereotypes/misperceptions, and attitudes. The total scales and their subscales demonstrated good internal reliability (alpha coefficients 0.72–0.94). The mean scores for each scale were significantly associated with each other, with related measures, and differed by sociodemographic characteristics. Specifically, in adjusted analyses, women in the university setting and White women expressed more negative attitudes and stigma around parenting. Minority women endorsed more negative norms and stigma around adoption. Finally, women from the health department, White women, and religious women expressed more negative norms and stigma around abortion.

**Conclusion:**

Findings suggest that our multidimensional measures have good psychometric properties in our sample of young women in the U.S. South, and highlight the importance of conceptualizing and measuring norms and stigmas around all pregnancy decisions. These scales may be of use in research on pregnancy decision-making and evaluation of stigma-reduction interventions.

## Introduction

Unintended pregnancy is a significant public health concern in the United States. While the national rate of unintended pregnancy decreased in the past decade, significant disparities remain [1]. The highest rates of unintended pregnancy occur in U.S. women who identify as minority, socioeconomically disadvantaged, less educated, unmarried, and younger (under 24 years of age). These populations also bear the greatest burden of adverse outcomes associated with unintended pregnancy, such as romantic relationship dissolution [2], substance use and suboptimal prenatal care utilization during pregnancy, as well as premature birth, low birthweight and lower perceived likelihood of breastfeeding for women who decide to proceed with childbirth [3, 4]. Despite the challenges and risks associated with unintended childbearing, abortion and adoption are less commonly selected alternatives in the U.S. [1, 5].

Young women and women in the U.S. South are more likely than older women and women in other regions to both experience an unintended pregnancy and carry that pregnancy to term [1, 6]. Young women are less likely to want a pregnancy at this stage in their lives [3], and may have less access to reproductive health care than their older counterparts [7]. At the same time, women in the South have less access to the full spectrum of reproductive health services than women in other parts of the country [8]. These factors contribute to the complexity with which women perceive their options during unintended pregnancy [9]. However, research on the role of attitudes regarding pregnancy decisions in decision-making processes during unintended pregnancy is limited. Recent studies hypothesizes that norms and stigma influence pregnancy decision-making processes during unintended pregnancy [10], particularly among young women in the U.S. South [11, 12].

Young women make pregnancy decisions considering norms within their social networks that dictate acceptable alternatives when making pregnancy decisions. Less socially accepted pregnancy decisions are subject to interpersonal manifestations of stigma such as negative attitudes and social judgment from others [11]. In Alabama, disparaging views of abortion may be more pronounced relative to parenting and adoption, given public opposition to abortion and the restrictive abortion service environment in the state [8, 13]. In a qualitative study, Smith, Turan and colleagues recently reported that young women in central Alabama perceive and endorse abortion stigma within their communities, perpetuated via social circles and local organizations (e.g., crisis pregnancy centers that discourage abortion and incentivize adoption and parenting). Though less studied, norms and stigma around young parenting and adoption are also salient among this population. Young women in Alabama have shared peer and community norms that support childbearing in late adolescence, in spite of negative social reactions to parenting outside of ideal socioeconomic and relationship conditions. Adoption is comparatively less common, and is socially viewed as a moral middle ground between parenting and abortion among young Alabama women [11].

Considering this social tendency to juxtapose pregnancy decisions with one another [14] and the personal utility of making pregnancy decisions that elicit approval from close others [15], young women in this setting may have limited reproductive autonomy. Prior literature hypothesizes that stigma regarding unintended pregnancy and pregnancy decisions may manifest in forms of reproductive coercion such as pressure to terminate a pregnancy, pressure to choose adoption over abortion, or pressure to continue with an unwanted pregnancy [11]. For example, young women who want to parent can experience coercion to hide a pregnancy by abortion or adoption due to social sanctions around parenting during emerging adulthood [11, 16]. The potential effects of these reproductive stigmas also extend beyond pregnancy decision-making to health care service utilization and adverse health outcomes.

When the continuation of an unwanted pregnancy is coerced, women are at risk of poor prenatal behaviors such as lack of prenatal care utilization, smoking and alcohol use, which have implications for birth outcomes such as low birthweight and perinatal mortality [17]. Conversely, among women seeking abortion, abortion stigma is associated with depressive symptoms, anxiety, and stress among women prior to abortion [18], as well as with whether and when women seek abortion care from a qualified health provider [16, 19]. While the current literature does not support the notion that abortion itself is related to poor long-term mental health outcomes [20, 21], manifestations of abortion stigma in the form of restricted access to abortion and secrecy surrounding having an abortion are associated with increased psychological distress [22, 23]. Relatively little evidence exists as to the potential effects of adoption and parenting stigma, however, perhaps because these concepts have yet to be quantified.

Considering findings from prior studies in this area, the levels of norms and stigma regarding each pregnancy decision (parenting, adoption, abortion) likely vary and may be differentially related to pregnancy decisions, healthcare utilization and adverse health outcomes. Measurement of these norms and stigmas may inform understanding of pregnancy decision-making among vulnerable young women. Researchers have developed and validated scales to measure abortion stigma in different contexts [24–26], but to our knowledge, no tools exist to measure norms and stigma around other pregnancy decisions among young women, and little is known about the social and demographic characteristics associated with variations in the content of norms regarding pregnancy decisions, and in stigma related to violations of these norms. In order to elucidate and quantify perceptions of reproductive stigma around a fuller range of reproductive options among young women, the present study sought to develop three distinct scales to measure norms and stigma around each potential pregnancy decision, and to identify predicting factors for each scale.

### Measurement of stigma regarding pregnancy decisions

Conceptualization of stigma in the reproductive health context, primarily from the abortion stigma literature, is complex. Frameworks have been developed to guide understanding of where stigma operates (e.g., at the level of individual, community, institution, etc.), who it affects (e.g., members of a stigmatized group, associated individuals, larger community, etc.), how it manifests (e.g., though experienced, perceived, anticipated, internalized and other forms of stigma), and how it is managed (e.g., though social distancing, secrecy, avoidance, etc.)[27, 28]. To date, abortion stigma scale developers have focused their measurement efforts on distinct components of these larger frameworks. For example, the Individual Level Abortion Stigma (ILAS) scale by Cockrill et al. consists of measures of self-judgment, anticipated judgment, perceived community condemnation, disclosure and perceived support among women who have had an abortion [25]. In contrast, the Stigmatizing Attitudes, Beliefs, and Actions Scale (SABAS) by Shellenberg et al. captures endorsement of negative stereotypes, discriminatory intentions, exclusivity, and fear of contagion regarding a woman who has had an abortion by community members, regardless of their personal abortion history [26].

The scales introduced in the current study similarly explore both interpersonal and intrapersonal manifestations of stigma at the individual level. Given that women are likely to form their perceptions of reproductive norms and stigmas prior to experiencing a pregnancy themselves [11], which may have bearing on how they experience an unintended pregnancy in the future or react to that of others, both women who have and have not had an unintended pregnancy are included in the present study. Therefore, self-stigma and management of stigma around prior pregnancy decisions were not measured. Rather, we investigated other dimensions of stigma, asking about attitudes toward pregnancy decisions and a hypothetical young woman who has chosen those pregnancy decisions.

## Methods

To achieve the study aims, we utilized an exploratory sequential mixed methods study design [29], which involved the use of a multiple stage process in which qualitative methods informed the development and testing of quantitative items that were subsequently assessed in a large sample of young adult women in Alabama. The current study protocol was approved by the University of Alabama at Birmingham (UAB) Institutional Review Board.

### Item development

Initially, 6 focus groups were conducted to develop an understanding of the constructs of norms and stigma from the perspective of low-income young adult women in the Alabama context. Details of our focus group methodology have been reported previously, and are summarized briefly here [11]. Thirty-four young women recruited from public health department clinics and a community college in Birmingham, AL participated in 6 focus groups that were stratified by race (3 with White women and 3 with Black women) and facilitated by race concordant female moderators. Findings suggested that norms manifest in beliefs regarding the social acceptability of parenting, adoption, or abortion following an unintended pregnancy. They also manifest in negative anticipated emotional reactions to those pregnancy decisions within close social networks. All pregnancy decisions were subject to some forms of stigma; however, young women who choose abortion and adoption are viewed in terms of more negative attitudes and stereotypes than those who choose to parent. Qualitative results were used as a framework to develop a pool of nearly 100 questionnaire items related to acceptable circumstances, anticipated reactions, stereotypes, and attitudes regarding each pregnancy decision and young women who make those decisions.

To assess face validity, we subsequently consulted with a group of experts inside and outside of the state with professional knowledge of direct service provision, research, and legal issues in reproductive health, and revised the questionnaire items based upon their feedback. To further refine the item pool and assess content validity, 12 cognitive interviews were conducted in a separate sample of young adult women in Alabama, in which participants assessed the items for comprehensibility and interpretability. The items and response options were then revised to improve content and clarity. The cognitive interview methodology that were utilized have also been reported previously [11]. Prior to implementation, the item pool plus demographic and health questions were programmed into iSurvey software, for conduct of self-administered iPad-based surveys [30]. The initial iPad version of the questionnaire was pretested with 12 female graduate student volunteers, and then revised to correct errors and improve formatting.

We tested the resulting 97 items in Birmingham, Alabama S1 File. The 97 items were separated into three sections of the survey by pregnancy decision. Fifty-two of the items measured agreement with stereotypes/misperceptions regarding or conditional acceptability of pregnancy decisions (e.g., “Women my age who decide to place the baby for adoption after becoming pregnant accidentally usually are not ready to be mothers”, “If a woman is going to keep the baby, she should have family support”, etc.) on a five-level bidirectional Likert scale (1 = “strongly disagree,” 2 = “disagree,” 3 = “neither agree nor disagree”, 4 = “agree,” 5 = “strongly agree”). Thirty items measured anticipated reactions to a pregnancy decision (e.g., “If I got pregnant accidentally and decided to have an abortion, the people who matter most to me would be mad”) by the frequency or perceived likelihood of the experience on a five-level bidirectional scale (e.g., 1 = “extremely unlikely,” 2 = “unlikely,” 3 = “neutral”, 4 = “likely,” 5 = “extremely likely”). Fifteen items measured attitudes towards young women who make certain pregnancy decisions (e.g., “how selfish is a woman your age who has the baby and raises it herself”) on a five-level unidirectional scale (1 = “not at all”, 2 = “a little bit”, 3 = “somewhat”, 4 = “quite”, 5 = “extremely”). In addition to the norms and stigma survey items, we included socio-demographic, health behavior and health outcome questions. The sociodemographic and health items included questions about the participants’ age, place of birth, length of residence in Birmingham, AL, household receipt of public assistance, race, religion, religiosity, and sexual and reproductive history (e.g., number of previous pregnancies and abortions, recent history of unintended pregnancy, outcome of most recent pregnancy, etc.).

### Survey implementation

Women aged 18–24 attending local public health department clinics or a local public university, who were English-speaking and not pregnant at the time of the study, were eligible to participate in the iPad survey. Multiple convenience sampling recruitment methods were used to enroll study participants. In one scenario, trained graduate research assistants approached potential participants within common spaces of the university and health centers (i.e., lobbies or waiting areas), provided a verbal explanation of the study, and screened women interested in participating for eligibility. In some cases, the clinic front desk staff directed potential participants to study staff, at which point the study staff proceeded to describe the study and screen young women for eligibility.

Additional participants attending the university were recruited through an Introduction to Psychology course student research participation system. Students in the course were required to participate in research for course credit, achieved either through involvement in a study of their choosing or by completion of a literature synthesis assignment. Students interested in study participation used an online system to view study descriptions, eligibility criteria, and contact information, as well as to sign up or cancel an appointment for the approved studies.

During study enrollment, trained research assistants provided eligible and interested women with an iPad on which to complete the survey. Participants began by reading the consent form on the iPad, and then proceeded to select “I consent” or otherwise on the iPad touch screen. After consenting, participants encountered the survey items followed by the demographic and health questions. Survey participants who were not in the Introduction to Psychology course were compensated $20 for participation, whereas students in the course were provided with documentation of research participation credit and a description of the relevance of the study aims to concepts learned within the course. All study participants were offered a counseling resource sheet and printout of the consent form at the conclusion of the survey.

### Analyses of scale properties and bivariate associations

We conducted exploratory principal components analyses to examine the structure of distinct scales to measure stigma associated with adoption, abortion and parenting following an unintended pregnancy. Three separate analyses were performed for adoption (30 items entered), abortion (34 items entered), and parenting (30 items entered), respectively. Parallel analysis was used to determine how many factors (also referred to herein as subscales) to retain [31, 32]. Items with negative factor loading and items with positively worded items were reverse-coded so that higher scores on each scale and subscale reflect more negative attitudes and stigma around that pregnancy decision.

We performed reliability analyses to determine the internal consistency of the resulting subscales. We eliminated subscales with Cronbach’s alpha under 0.70, and items which would reduce the subscale or total scale reliability by Cronbach’s alpha of 0.02 points or more. Some of the sub-scales consisted of few items, but were retained on the basis of theoretical importance, as well as evidence of internal consistency. Separate principal components analyses were performed among pregnancy history, race, recruitment site, and religiosity subgroups, and these results were compared with the results of principal components analyses within the full sample.

Correlations between the scores for each scale and subscale (the mean of the items comprising a scale or subscale) and measures of constructs that the subscales would be expected to be correlated with were examined in order to examine construct validity. For this purpose, three items from the survey that indicated how likely participants perceived that they would be to parent a child, place a child for adoption, or have an abortion following an unintended pregnancy were selected (e.g., “Imagine that you just found out you became pregnant by accident. How likely would you be to do the following: [pregnancy decision]?”). Items were measured on a five-point bidirectional scale from “extremely unlikely” to “extremely likely”. We hypothesized that the parenting norms and stigma subscales and total scale should correlate negatively with perceived likelihood of parenting and positively with perceived likelihood of adoption and abortion if faced with an unintended pregnancy. We hypothesized that the adoption norms and stigma subscales and the total scale should negatively correlate with perceived likelihood of parenting and adoption, and positively with perceived likelihood of abortion in our sample. Lastly, we hypothesized that the abortion stigma scale and subscales should correlate negatively with perceived likelihood of abortion and positively *w*ith the perceived likelihood of parenting or adoption.

We also examined whether total scale scores (also treated as mean score variables) for each pregnancy decision were correlated with one another to explore bivariate relationships among the scales. T-tests to explore mean differences in scores on the total scales by participant characteristics were subsequently performed to identify whether any subgroups reported less versus more agreement with norms and stigma statements. To determine whether the bivariate relationships observed remained when controlling for other characteristics, multiple linear regression analyses examining predictors of total scale scores were performed. We used Stata 13 to conduct the parallel analyses and SPSS 22 for all other analyses.

## Results

### Sample characteristics

Six hundred and forty-two young adult women completed the iPad survey, of which the majority were 18–19 years of age (Table 1). Around 70% of participants were recruited from the university campus as opposed to the health department site. Just over 45% of the sample was Black, approximately 40% White, and 15% of other race/ethnicity. A substantial proportion of participants (38%) reported that someone in their household received public assistance within the last month. Thirty-five percent of women identified as being very religious or spiritual. Seventeen percent had experienced at least one pregnancy, amongst which approximately half reported their most recent pregnancy as being unintended and over half reported the decision to parent after that recent pregnancy.

**Table 1:** Demographic characteristics of young adult women in Birmingham, AL who participated in the iPad survey (n = 642).

Participant characteristics	% (n) or Range, M (SD)
Age	18–24, 19.91 (2.02)
Recruitment Site	
Health department	28.2 (181)
University	72.8 (461)
Race	
Black	45.5 (290)
White	39.5 (252)
Other*	15.0 (96)
Household Public Assistance	
Received in Past Month	37.7 (242)
Not Received in Past Month	62.3 (400)
Religiosity/Spirituality	
Very Religious/Spiritual	34.6 (222)
Not or Somewhat Religious/Spiritual	65.4 (420)
Relationship Status	
Single	54.0 (347)
Not Single	46.0 (295)
Prior Pregnancies (n = 636)	
1 or more	17.0 (105)
None	83.0 (531)
Pregnancy Intendedness (n = 105)†	
Unintended	51.0 (54)
Not Unintended	49.0 (51)
Prior Pregnancy Decision (n = 105)±	
Parenting	53.0 (56)
Adoption	3.0 (3)
Abortion	27.0 (28)
Miscarriage	17.0 (18)

*Note*: Number of responses indicated when the number of responses differs from the entire sample due to missing data or survey skip pattern

* Other includes American Indian/Alaskan Native, Asian, Native Hawaiian/Other Pacific Islander, or any other racial category not listed

†Participants reporting a prior pregnancy responded to the question, “Regarding your most recent pregnancy, did you get pregnant sooner than you wanted, or at a time when you did not want to get pregnant at all?”

± Refers to outcome of the participants’ most recent pregnancy.

### Scale properties

#### Parenting scale

The parallel analysis yielded six factors for retention in the principal components analysis for parenting, which accounted for 53.10% of the variance in the scale. Factor 1 reflected anticipated reactions by close others to the decision to parent during an unintended pregnancy, represented 17.63% of the variance, and had an eigenvalue of 5.29. Factor 2 reflected attitudes about a young woman who makes the decision to parent, and it represented 10.12% of the variance, and had an eigenvalue of 3.04. Factor 3 represented conditions upon which it is socially acceptable for a young woman to elect to parent during an unintended pregnancy (8.62% variance explained; eigenvalue = 2.59). Factor 4 represented stereotypes about young women who choose to parent during an unintended pregnancy (6.56% variance explained; eigenvalue 1.97). Table 2 presents the items retained following internal reliability analysis for the four parenting subscales. The remaining factors had low internal reliability (alpha < 0.70), and thus were excluded from the total scale.

**Table 2:** Item factor loadings, mean (standard deviations), and proportions for the parenting norms and stigma scale (n = 642).

Subscales and Items	Factor loading	alpha	M (SD)
Conditional Acceptability Subscale (5 items)		0.72	2.13 (0.65)
If a woman is going to keep the baby, she should be in a committed relationship.†	0.56		3.29 (1.15)
If a woman is going to keep the baby, she should have family support.†	0.68		2.09 (1.03)
If a woman is going to keep the baby, she should have a job.†	0.68		1.73 (0.81)
If a woman is going to keep the baby, she should have a stable place to live.†	0.74		1.43 (0.66)
If a woman is going to keep the baby, she should have at least a high school degree.†	0.70		2.10 (1.03)
Anticipated Reactions Subscale (6 items)		0.89	2.91 (0.88)
If you became pregnant accidentally, and decided to keep the baby, how likely is it that the people who matter most to you would feel the following ways:			
Would be disappointed	0.84		2.87 (1.45)
Would be happy†	0.79		2.59 (1.19)
Would be mad	0.87		2.77 (1.31)
Would understand†	0.78		2.39 (1.16)
Would be supportive†	0.62		4.31 (0.94)
Would be ashamed	0.67		2.51 (1.35)
Stereotypes Subscale (4 items)		0.72	3.08 (0.77)
Women my age who keep the baby after becoming pregnant accidentally:			
Are usually low income	0.66		3.34 (1.05)
Usually are trying to keep their man	0.65		2.86 (1.09)
Usually are not well educated	0.73		2.83 (1.02)
Usually end up on welfare	0.81		3.29 (1.04)
Attitudes Subscale (5 items)		0.74	1.32 (0.54)
In your opinion, how irresponsible is a woman your age who has the baby and raises it herself?	0.51		1.42 (0.92)
In your opinion, how mature is a woman your age who has the baby and raises it herself?†	0.67		1.55 (0.88)
In your opinion, how selfish is a woman your age who has the baby and raises it herself?	0.81		1.26 (0.72)
In your opinion, how strong is a woman your age who has the baby and raises it herself?†	0.77		1.25 (0.68)
In your opinion, how cold‎/heartless is a woman your age who has the baby and raises it herself?	0.77		1.13 (0.53)
Total Parenting Norms and Stigma Scale (20 items)		0.73	2.23 (0.41)

Note

† represents reverse coding.

#### Adoption scale

Parallel analysis for adoption items yielded five factors, accounting for 53.82% of the variance in the scale. Factor 1 reflected attitudes about a young woman who makes the decision to adopt, represented 24.59% of the variance, and had an eigenvalue of 7.38. Factor 2 reflected anticipated reactions by close others to the decision to place their child for adoption during an unintended pregnancy, and it represented 10.90% of the variance, and had an eigenvalue of 3.27. Factor 3 represented stereotypes about young women who choose to place a child for adoption following unintended pregnancy (7.36% variance explained; eigenvalue = 2.21). Factor 4 represented accepted conditions upon which a young woman can choose adoption during an unintended pregnancy (5.85% variance explained; eigenvalue 1.75). Table 3 presents the items retained following internal reliability analysis for the four adoption subscales. The remaining factor had insufficient internal reliability (alpha < 0.70), and thus was excluded from the total scale.

**Table 3:** Item factor loadings, mean (standard deviations), and proportions for the adoption norms and stigma scale (n = 642).

Subscales and Items	Factor loading	alpha	M (SD)
Conditional Acceptability Subscale (2 items)		0.76	2.57 (1.05)
If your family will not support you in having a baby, it’s okay to place your child for adoption.†	0.68		2.32 (1.12)
If the man involved in the pregnancy will not support you in having a baby, it’s okay to place your child for adoption.†	0.58		2.83 (1.21)
Anticipated Reactions Subscale (7 items)		0.87	3.30 (0.93)
If I got pregnant accidentally, and decided to place the baby for adoption, the people who matter most to me:			
Would be disappointed.	0.72		3.59 (1.26)
Would be happy.†	0.74		3.60 (1.13)
Would be mad.	0.77		3.38 (1.26)
Would be surprised.	0.47		3.92 (1.10)
Would understand.†	0.80		2.94 (1.34)
Would be supportive.†	0.80		2.67 (1.32)
Would feel ashamed.	0.58		2.98 (1.35)
Stereotypes Subscale (4 items)		0.78	3.91 (0.70)
Women my age who decide to place the baby for adoption after becoming pregnant accidentally:			
Are usually low income.	0.79		3.55 (1.03)
Usually are not in a committed relationship.	0.83		3.69 (0.93)
Usually do not have family support to raise the child.	0.82		3.98 (0.88)
Usually are not ready to be mothers.	0.61		4.41 (0.73)
Attitudes Subscale (6 items)		0.88	2.41 (0.97)
Women who place a baby for adoption regret it later.	0.44		3.36 (0.98)
In your opinion, how irresponsible is a woman your age who places her baby for adoption?	0.81		2.09 (1.28)
In your opinion, how mature is a woman your age who places her baby for adoption?†	0.75		2.49 (1.20)
In your opinion, how selfish is a woman your age who places her baby for adoption?	0.84		2.25 (1.29)
In your opinion, how strong is a woman your age who places her baby for adoption?†	0.73		2.15 (1.27)
In your opinion, how cold/heartless is a woman your age who places her baby for adoption?	0.84		2.16 (1.29)
Total Parenting Norms and Stigma Scale (20 items)		0.73	2.23 (0.41)

Note

† Reverse coded

#### Abortion scale

Analysis of abortion items yielded four factors, accounting for 52.84% of the variance in the scale. Factor 1 reflected both attitudes about a young woman who makes the decision to have an abortion and conditions upon which it is acceptable for a young woman to have an abortion following an unintended pregnancy. Factor 1 represented 31.72% of the variance, and had an eigenvalue of 10.79. Given the prior findings that attitudes and conditional acceptability were separate factors, and thus considered separate constructs, in the parenting and adoption scales, we entered the two into reliability analyses separately. The Cronbach’s alpha for the two subscales were sufficient to be considered as separate (Table 4). Factor 2 reflected anticipated reactions by close others to the decision to have an abortion following an unintended pregnancy, and it represented 9.70% of the variance, and had an eigenvalue of 3.30. Factor 3 represented misperceptions about the medical risk of abortion for young women who choose to have an abortion following unintended pregnancy (6.25% variance explained; eigenvalue = 2.12). The remaining factor had insufficient internal reliability (alpha < 0.70), and thus was excluded from the total scale.

**Table 4:** Item factor loadings, mean (standard deviations), and proportions for the abortion norms and stigma scale (n = 642).

Subscales and Items	Factor loading	alpha	M (SD)
Conditional Acceptability Scale (4 items)		0.94	3.49 (1.20)
Abortion is acceptable if the woman cannot take care of her child.†	0.86		3.39 (1.38)
Abortion is acceptable if the woman does not have family support.†	0.84		3.45 (1.30)
If the man involved in the pregnancy will not support you in having a baby, it's okay to have an abortion.†	0.82		3.73 (1.22)
If your life is really messed up, it's better to have an abortion than to keep the baby.†	0.82		3.40 (1.34)
Anticipated Reactions Scale (7 items)		0.88	4.02 (0.94)
If I got pregnant accidentally and decided to have an abortion, the people who matter most to me:			
Would be disappointed.	0.78		4.33 (1.07)
Would be happy.†	0.75		4.31 (1.07)
Would be mad.	0.79		4.08 (1.19)
Would be surprised.	0.48		4.28 (1.05)
Would understand. †	0.78		3.83 (1.33)
Would be supportive.†	0.75		3.69 (1.42)
Would feel ashamed.	0.62		3.62 (1.45)
Misperceptions Scale (2 items)		0.81	4.06 (0.99)
Abortion is risky for women’s health.	0.79		4.04 (1.10)
Women who have multiple abortions may not be able to have a child later in life.	0.84		4.09 (1.06)
Attitudes Scale (8 items)		0.90	3.26 (1.09)
Abortion is acceptable in any situation.†	0.71		3.95 (1.16)
Women who have abortions are killing their own children.	0.59		3.70 (1.36)
Abortion should be the woman’s decision.†	0.60		2.38 (1.35)
In your opinion, how irresponsible is a woman your age who has an abortion.	0.75		3.07 (1.44)
In your opinion, how mature is a woman your age who has an abortion.†	0.74		3.52 (1.30)
In your opinion, how selfish is a woman your age who has an abortion.	0.77		3.39 (1.49)
In your opinion, how strong is a woman your age who has an abortion.†	0.68		2.98 (1.52)
In your opinion, how cold/heartless is a woman your age who has an abortion.	0.78		3.11 (1.55)
Total Abortion Norms and Stigma Scale (21 items)		0.94	3.63 (0.86)

Note

† indicates reverse coding.

#### Sensitivity analyses

Across pregnancy decisions, when we compared principal components analyses conducted among the full sample to principal components analyses conducted among sub-groups (by pregnancy history, race, recruitment site, and religiosity), we found that for each sub-group, the items entered into analysis consistently grouped together into the same principal components that comprise the sub-scales for norms and stigma regarding pregnancy decisions (i.e., anticipated reactions, attitudes, conditional acceptability, and stereotypes regarding each pregnancy decision).

### Bivariate associations

Most of the correlations were in the expected directions, but unexpected results were also observed (see Table 5). All parenting stigma subscales and the total scale were negatively correlated with perceived likelihood of parenting and positively correlated with perceived likelihood of abortion and adoption. In contrast, the adoption and abortion subscales and total scales were negatively correlated with perceived likelihood of adoption and abortion, but positively correlated with perceived likelihood of parenting. Additional correlation analysis (not shown) indicated that women who scored higher on the parenting norms and stigma total scale scored lower on the adoption (*r* = -0.16, p<.001) and abortion scales (*r* = -0.19, p <.001). The adoption stigma and abortion stigma scales were positively correlated (*r* = 0.40, p<.001). Overall, these analyses support construct validity for our stigma measures, in that women who have high levels of stigma around a certain decision (e.g., abortion) report less perceived likelihood that they would choose that option if faced with an unintended pregnancy (e.g., choose to abort), and higher perceived likelihood that they would choose alternative options (e.g., parenting).

**Table 5:** Bivariate correlations between mean scores for abortion, adoption, and parenting norms and stigma scale items and perceived likelihood of pregnancy decisions (N = 642).

	Conditional Acceptability	Anticipated Reactions	Stereotypes/ Misperceptions	Attitudes	Total Scale
Parenting Norms and Stigma Subscales and Total Scale					
Perceived Likelihood of Parenting	-0.48	-0.29***	-0.59	-0.15***	-0.34***
Perceived Likelihood of Adoption	0.10**	0.20***	0.23	0.55	0.21**
Perceived Likelihood of Abortion	0.05	0.25***	0.08*	0.11**	0.30***
Adoption Norms and Stigma Subscales and Total Scale					
Perceived Likelihood of Parenting	0.33***	0.29***	0.04	0.29***	0.35***
Perceived Likelihood of Adoption	-0.41***	-0.46***	-0.04	-0.39***	-0.50***
Perceived Likelihood of Abortion	-0.29***	-0.18***	-0.02	-0.21***	-0.24***
Abortion Norms and Stigma Subscales and Total Scale					
Perceived Likelihood of Parenting	0.57***	0.34***	0.32***	0.51***	0.56***
Perceived Likelihood of Adoption	-0.41	0.03	-0.12**	-0.08*	-0.05
Perceived Likelihood of Abortion	-0.73***	-0.47***	-0.31***	-0.66***	-0.72***

Note

* denotes significant correlation at the 0.05 level

** denotes p<0.01 level

*** denotes p<0.001 (2-tailed).

The exploration of differences in the total scale scores according to characteristics of the young women who participated in the study revealed different patterns for each scale (see Table 6). Significant differences in the parenting scale scores were observed by recruitment site, relationship status, and religion, with those women who were from the university, single, and nulliparous expressing more negative norms and stigma around parenting. Significant differences in the adoption scale were observed for several demographic characteristics. Women who were recruited from the health department, Black, living in household where public assistance was recently received, not single, as well as women with a pregnancy history, and whose most recent pregnancy resulted in parenting and did not result in abortion were in more agreement with negative norms and stigma around adoption. Significant differences for the abortion stigma scale were observed by recruitment site, degree of religiosity/spirituality and recent reproductive history, with those who were from the health department, identified as very religious or spiritual, had a recent unintended pregnancy, and whose recent pregnancy did not result in an abortion expressing more negative norms and stigma related to abortion.

**Table 6:** Characteristics associated with variation in total parenting, adoption, and abortion norms and stigma mean scores in bivariate analyses (n = 642).

Participant characteristics	Total Parenting Scale, Mean (S.D.)	Total Adoption Scale, Mean (S.D.)	Total Abortion Scale, Mean (S.D.)
Recruitment Site	**p<.001**	**p<.001**	**p = 0.35**
Health department	2.05 (0.41)	3.49 (0.62)	3.75 (0.75)
University	2.30 (0.39)	2.90 (0.60)	3.59 (0.89)
Race	p = 0.23	**p<.001**	p = 0.45
Black	2.18 (0.43)	3.29 (0.64)	3.66 (0.74)
White	2.22 (0.39)	2.82 (0.62)	3.71 (0.94)
Household Public Assistance	p = 0.32	**p<.001**	p = 0.42
Received in Past Month	2.21 (0.45)	3.23 (0.65)	3.67 (0.81)
Not Received in Past Month	2.25 (0.39)	2.97 (0.65)	3.61 (0.88)
Religiosity/Spirituality	p = 0.27	p = 0.19	**p<.001**
Very Religious/Spiritual	2.21 (0.44)	3.12 (0.71)	3.96 (0.80)
Not or Somewhat Religious/Spiritual	2.25 (0.40)	3.05 (0.64)	3.46 (0.84)
Relationship Status	**p = 0.02**	**p = 0.03**	p = 0.82
Single	2.27 (0.39)	3.02 (0.69)	3.63 (0.85)
Not Single	2.19 (0.44)	3.13 (0.63)	3.64 (0.87)
Prior Pregnancies (n = 636)	**p<.001**	**p<.001**	p = 0.99
1 or more	2.10 (0.40)	3.40 (0.63)	3.64 (0.79)
None	2.26 (0.41)	3.01 (0.65)	3.64 (0.87)
Pregnancy Intendedness (n = 105)†	p = 0.42	p = 0.05	**P = 0.02**
Unintended	2.07 (0.36)	3.52 (0.65)	3.82 (0.71)
Not Unintended	2.13 (0.41)	3.28 (0.59)	3.47 (0.82)
Parenting (n = 105)±	p = 0.67	**p = <0.01**	**p<.001**
Yes	2.02 (0.39)	3.59 (0.66)	4.02 (0.67)
No	2.17 (0.38)	3.22 (0.55)	3.31 (0.75)
Abortion (n = 105)±	p = 0.95	**p<0.01**	**p<.001**
Yes	2.10 (0.35)	3.11 (0.45)	3.05 (0.60)
No	2.11 (0.41)	3.50 (0.65)	3.86 (0.73)

*Note*: Higher scores indicate higher stigma; S.D. represents standard deviation; Number of responses indicated when the number of responses differs from the entire sample due to missing data or survey skip pattern

†Participants reporting a prior pregnancy responded to the question, “Regarding your most recent pregnancy, did you get pregnant sooner than you wanted, or at a time when you did not want to get pregnant at all?” refers to most recent pregnancy

± Refers to outcome of the participants’ most recent pregnancy.

### Multivariate associations

Multivariate linear regression analyses revealed that recruitment site was associated with differences in each of the total scales (Table 7), even after adjusting for the other predictors. Participants from the health department scored lower than university participants on the parenting stigma scale score, while controlling for other covariates, but the inverse was true for the adoption and abortion norms and stigma scales. In adjusted analyses, race was also associated with differences on all scales, whereby Black women scored higher on the adoption norms and stigma scale compared to White women, and women who identified as “Other” race scored higher on the parenting and adoption norms and stigma scales as well as lower on the abortion norms and stigma scale, relative to White women. Lastly, women who are very religious/spiritual score higher on the abortion norms and stigma scale than women who are not at all or somewhat religious/spiritual.

**Table 7:** Demographic predictors of variation in mean parenting, adoption, and abortion norms and stigma scale scores in multivariate linear regression analyses (n = 636).

Participant characteristics	Total Parenting Scale, *B* (*SE*)	Total Adoption Scale, *B* (*SE*)	Total Abortion Scale, *B* (*SE*)
Recruitment Site			
Health department	-0.26 (0.45)***	0.51 (0.07)***	0.20 (0.09)*
University	ref	ref	ref
Race			
Black	0.02 (0.38)	0.29 (0.06)***	-0.13 (0.08)
White	ref	ref	ref
Other	0.16 (0.05)**	0.28 (0.07)***	-0.34 (0.10)**
Household Public Assistance			
Received in Past Month	0.05 (0.04)	0.06 (0.05)	0.05 (0.07)
Not Received in Past Month	ref	ref	ref
Religiosity/Spirituality			
Very Religious/Spiritual	-0.03 (0.03)	0.09 (0.05)	0.46 (0.07)***
Not or Somewhat Religious/Spiritual	ref	ref	ref
Relationship Status			
Single	0.03 (0.03)	-0.07 (0.05)	0.01 (0.07)
Not Single	ref	ref	ref
Prior Pregnancies			
1 or more	0.01 (0.05)	-0.05 (0.08)	-0.10 (0.11)
None	ref	ref	ref

Note

*B* represents the unstandardized beta; *SE* represents standard error

* denotes significant correlation at the 0.05 level

** denotes p<0.01 level

*** denotes p<0.001 (2-tailed).

## Discussion

The present study developed scales to measure norms and stigma regarding parenting, adoption and abortion following unintended pregnancies, each with four dimensions: conditional acceptability, anticipated reactions, stereotypes, and attitudes. The scales and their subscales demonstrated good psychometric properties in a sample of young adult women in Birmingham, Alabama. In the current sample, mean responses to the total parenting scale (indicating the level of expression of negative attitudes and stigma) were lowest, followed by the adoption scale, and subsequently the abortion scale. The mean scores for each scale also demonstrated distinct relationships with each other, with other related measures of perceived behavioral intention, and differed by demographic characteristics. This highlights the importance of conceptualizing and measuring norms and stigmas around all pregnancy decisions, not abortion exclusively. In reality, women do not make abortion decisions in a vacuum where they only think about abortion, but rather in the context of their other pregnancy options.

In the current study, more negative attitudes and stigma around a given pregnancy decision were negatively correlated with participants’ perceived likelihood of choosing that specific pregnancy decision if faced with an unintended pregnancy. Greater score on both the total scale and the subscales for abortion, adoption, or parenting norms and stigma was associated with lower perceived likelihood of abortion, adoption, or parenting in bivariate analyses, respectively, as we had expected. Thus, the theoretical construct of norms and stigma regarding pregnancy decisions that we intended to measure does indeed appear to reflect negative affect regarding those decisions. We also observed an inverse relationship between the parenting scale and subscale scores, and the scores for, as well as the perceived likelihood of adoption or abortion. This finding provides support for prior studies that report that perceptions of abortion and adoption among young women in the U.S. South are grounded in social norms around motherhood [11, 12].

Our findings additionally suggest that specific demographic predictors may be related to expression of negative norms and stigma around certain pregnancy decisions. For example, very spiritual/religious young women in our sample score higher on the abortion scale, as compared to women who are not at all or somewhat religious. This result mirrors that of previous abortion stigma scale development studies [24, 25]. We also report that Black women and other non-White women scored higher than White women on the adoption scale, which may reflect implicit or explicit perceptions of racial biases in U.S. adoption system and their persistent impact on minority children placed for adoption [33, 34]. The present study results also indicate that women recruited from the university setting, an environment in which young women may be prone to delay parenting until later [35], endorsed more negative attitudes and stigma around parenting and less around other pregnancy decisions in the face of an unintended pregnancy. Existing literature similarly indicates that differences in social opportunity, including higher education, correspond with norms around pregnancy timing and pregnancy decision-making [36].

In the present study, women whose most recent pregnancy resulted in abortion had lower mean scores on the abortion scale than women who had a recent pregnancy, but did not have an abortion. This finding complements results by Cockrill et al., that women in their sample of participants who had an abortion perceived abortion stigma on the low range of possible scale and subscales responses [25]. The social phenomena operating here in which women who make a pregnancy decision report less negative attitudes regarding the pregnancy decision that they themselves have chosen, but more negative norms and stigma regarding other decisions, may also explain our finding that those who had a recent pregnancy and decided to parent score express more negative adoption and abortion attitudes. Women who make any pregnancy decision may feel validated by endorsement of negative attitudes regarding other pregnancy decisions. Furthermore, due to social desirability bias and the stigmatized nature of abortion procedures, the self-report measurement of abortion is subject to underreporting. The issue of underreporting due to stigma complicates our understanding of these findings, as women who feel the greatest amount of stigma around their decision would be less likely to report having had an abortion [25].

### Limitations and future directions

Knowledge of the specific groups more likely to endorse negative attitudes regarding pregnancy decisions is pertinent to efforts to address persistent inequalities in maternal health. The implications of our study should be understood in the context of its limitations, however. First, the results of the present study cannot be generalized to all young women living in the U.S. Women ages 18 to 24 in our sample who were recruited from health department clinics and a public university in the Birmingham, Alabama metro area represented a diverse group based upon several sociodemographic characteristics, but might be inherently different from other women who do not utilize healthcare services, attend a university, and reside in other areas of the state/country. While the current study was intended to provide an understanding of the southern U.S. context for young women, future studies should examine whether the pregnancy decision scales and their subscales are valid in other samples of women and men, including those in other regions and age groups.

Another possible limitation is selection bias. Women with more socially liberal views may have been more likely to participate, given the study topic and given the inclusion of a large number of university women in the study. Our strategy of recruitment from sites where the sociodemographic representation varies may have minimized this sort of selection bias. Additionally, we examined the few missing responses in our data and found that there was minimal potential for non-response bias. Nevertheless, if such non-response bias was present, our non-representative sample could have resulted in measurement error within the scale development and validation processes for the current study. Furthermore, selection bias would have reduced the effect sizes of our analyses due to limited variability of response (i.e., less extreme responses to the norms and stigma items), and made it more difficult to find associations between demographic predictors and variation in norms and stigma scale scores. Future research may use the reproductive norms and stigma scales developed in our study to understand the relationship between degree of reproductive norms and stigma, and reproductive coercion, pregnancy decision-making, other health behavior, or adverse maternal and child health outcomes. These subsequent studies should consider the use of simple or stratified random sampling strategies to reduce sampling error.

Relatedly, our study is potentially subject to social desirability bias as well. Although the surveys were self-administered on iPads, participants still may have been less likely to report more stigmatizing or non-normative beliefs in their responses to the norms and stigma items regarding pregnancy decisions. Additionally, measurement of abortion history in particular is subject to underreporting, particularly among women who report more stigma [25]. The use of self-administered electronic surveys was intended to facilitate greater accuracy in reporting on these sensitive topics through increased privacy.

This study represents the first psychometric evaluation of multi-dimensional scales of norms and stigma around all potential pregnancy decisions. Our findings underscore the significance of measuring attitudes regarding parenting, adoption, and abortion, particularly among neglected populations such as young women in the U.S. Deep South. Considering that reproductive norms and stigma can act to limit women’s reproductive autonomy and have negative effects on maternal and infant health, it is important that we monitor and address their pervasiveness using valid measures. Scales developed within the present study can be used in research examining the role of reproductive stigmas in pregnancy decision-making. Given that prior pregnancy decisions may affect subsequent responses to reproductive norms and stigma scale items, future studies should consider longitudinally measuring the relationships between reproductive stigma and future pregnancy decisions among women with a variety of prior pregnancy experiences. The measures presented here may also be used in evaluation of programs and interventions that aim to reduce unintended pregnancy, parenting, adoption, and/or abortion stigma.

## Supporting information

S1 File (DOCX)
