# Supplementary material for: Norms and stigma regarding pregnancy decisions during an unintended pregnancy: Development and predictors of scales among young women in the U.S. South
Source: PLoS One. 2017 Mar 22;12(3):e0174210. doi: 10.1371/journal.pone.0174210 (PMC5362217; doi:10.1371/journal.pone.0174210)
Supplement: S1 File — (DOCX) [file pone.0174210.s001.docx]

**Pool of Survey Items Entered into Analysis by Pregnancy Decision**

| **Parenting Items** |  |  | |  |
| --- | --- | --- | --- | --- |
| 1. In your opinion, how IRRESPONSIBLE is a woman your age who has the baby and raises it herself: | ☐ Not at all irresponsible  ☐ A little bit irresponsible  ☐ Somewhat irresponsible  ☐ Quite irresponsible  ☐ Extremely irresponsible | 1. In your opinion, how MATURE is a woman your age who has the baby and raises it herself: | ☐ Not at all mature  ☐ A little bit mature  ☐ Somewhat mature  ☐ Quite mature  ☐ Extremely mature | |
| 1. In your opinion, how SELFISH is a woman your age who has the baby and raises it herself: | ☐ Not at all selfish  ☐ A little bit selfish  ☐ Somewhat selfish  ☐ Quite selfish  ☐ Extremely selfish | 1. In your opinion, how STRONG is a woman your age who has the baby and raises it herself: | ☐ Not at all strong  ☐ A little bit strong  ☐ Somewhat strong  ☐ Quite strong  ☐ Extremely strong | |
| 1. In your opinion, how COLD/HEARTLESS is a woman your age who has the baby and raises it herself: | ☐ Not at all cold/heartless  ☐ A little bit cold/heartless  ☐ Somewhat cold/heartless  ☐ Quite cold/heartless  ☐ Extremely cold/heartless |  |  | |
| **Women my age who KEEP THE BABY after becoming pregnant accidentally:** | | | | |
| 1. are usually low income | ☐ Strongly disagree  ☐ Disagree  ☐ Neither agree nor disagree  ☐ Agree  ☐ Strongly agree | 1. can usually get family support to raise the child | ☐ Strongly disagree  ☐ Disagree  ☐ Neither agree nor disagree  ☐ Agree  ☐ Strongly agree | |
| 1. usually are trying to keep their man | ☐ Strongly disagree  ☐ Disagree  ☐ Neither agree nor disagree  ☐ Agree  ☐ Strongly agree | 1. are usually in a committed relationship | ☐ Strongly disagree  ☐ Disagree  ☐ Neither agree nor disagree  ☐ Agree  ☐ Strongly agree | |
| 1. usually have a decent job | ☐ Strongly disagree  ☐ Disagree  ☐ Neither agree nor disagree  ☐ Agree  ☐ Strongly agree | 1. usually are not well educated | ☐ Strongly disagree  ☐ Disagree  ☐ Neither agree nor disagree  ☐ Agree  ☐ Strongly agree | |
| 1. are usually ready to be a mother | ☐ Strongly disagree  ☐ Disagree  ☐ Neither agree nor disagree  ☐ Agree  ☐ Strongly agree | 1. usually end up on welfare | ☐ Strongly disagree  ☐ Disagree  ☐ Neither agree nor disagree  ☐ Agree  ☐ Strongly agree | |
| 1. are usually not responsible enough to go for checkups during pregnancy | ☐ Strongly disagree  ☐ Disagree  ☐ Neither agree nor disagree  ☐ Agree  ☐ Strongly agree |  |  | |
| **If you became pregnant accidentally and decided to KEEP THE BABY, how likely is it that the people who matter most to you:** | | | | |
| 1. would be disappointed | ☐ Extremely unlikely  ☐ Unlikely  ☐ Neutral  ☐ Likely  ☐ Extremely likely | 1. would be happy | ☐ Extremely unlikely  ☐ Unlikely  ☐ Neutral  ☐ Likely  ☐ Extremely likely | |
| 1. would be mad | ☐ Extremely unlikely  ☐ Unlikely  ☐ Neutral  ☐ Likely  ☐ Extremely likely | 1. would be surprised | ☐ Extremely unlikely  ☐ Unlikely  ☐ Neutral  ☐ Likely  ☐ Extremely likely | |
| 1. would understand | ☐ Extremely unlikely  ☐ Unlikely  ☐ Neutral  ☐ Likely  ☐ Extremely likely | 1. would not care | ☐ Extremely unlikely  ☐ Unlikely  ☐ Neutral  ☐ Likely  ☐ Extremely likely | |

| 1. would be concerned about me | ☐ Extremely unlikely  ☐ Unlikely  ☐ Neutral  ☐ Likely  ☐ Extremely likely | 1. would be supportive | ☐ Extremely unlikely  ☐ Unlikely  ☐ Neutral  ☐ Likely  ☐ Extremely likely |
| --- | --- | --- | --- |
| 1. would feel ashamed | ☐ Strongly disagree  ☐ Disagree  ☐ Neither agree nor disagree  ☐ Agree  ☐ Strongly agree |  |  |
| **If a woman is going to KEEP THE BABY, she should:** | | | |
| 1. be in a committed relationship | ☐ Strongly disagree  ☐ Disagree  ☐ Neither agree nor disagree  ☐ Agree  ☐ Strongly agree | 1. have family support | ☐ Strongly disagree  ☐ Disagree  ☐ Neither agree nor disagree  ☐ Agree  ☐ Strongly agree |
| 1. have a job | ☐ Strongly disagree  ☐ Disagree  ☐ Neither agree nor disagree  ☐ Agree  ☐ Strongly agree | 1. have a stable place to live | ☐ Strongly disagree  ☐ Disagree  ☐ Neither agree nor disagree  ☐ Agree  ☐ Strongly agree |
| 1. at least have a high school degree | ☐ Strongly disagree  ☐ Disagree  ☐ Neither agree nor disagree  ☐ Agree  ☐ Strongly agree | 1. I understand why some women get pregnant accidentally. | ☐ Strongly disagree  ☐ Disagree  ☐ Neither agree nor disagree  ☐ Agree  ☐ Strongly agree |
| 1. It is acceptable for a woman to raise a child by herself without a man around. | ☐ Strongly disagree  ☐ Disagree  ☐ Neither agree nor disagree  ☐ Agree  ☐ Strongly agree | 1. Imagine that you just found out you became pregnant by accident. How likely would you be to do the following: keep the baby and raise it myself? | ☐ Extremely unlikely  ☐ Unlikely  ☐ Neutral  ☐ Likely  ☐ Extremely likely |

| **Adoption Items** |  |  | |  |
| --- | --- | --- | --- | --- |
| 1. In your opinion, how IRRESPONSIBLE is a woman your age who places her baby for adoption: | ☐ Not at all irresponsible  ☐ A little bit irresponsible  ☐ Somewhat irresponsible  ☐ Quite irresponsible  ☐ Extremely irresponsible | 1. In your opinion, how MATURE is a woman your age who places her baby for adoption: | ☐ Not at all mature  ☐ A little bit mature  ☐ Somewhat mature  ☐ Quite mature  ☐ Extremely mature | |
| 1. In your opinion, how SELFISH is a woman your age who places her baby for adoption: | ☐ Not at all selfish  ☐ A little bit selfish  ☐ Somewhat selfish  ☐ Quite selfish  ☐ Extremely selfish | 1. In your opinion, how STRONG is a woman your age who places her baby for adoption: | ☐ Not at all strong  ☐ A little bit strong  ☐ Somewhat strong  ☐ Quite strong  ☐ Extremely strong | |
| 1. In your opinion, how COLD/HEARTLESS is a woman your age who places her baby for adoption: | ☐ Not at all cold/heartless  ☐ A little bit cold/heartless  ☐ Somewhat cold/heartless  ☐ Quite cold/heartless  ☐ Extremely cold/heartless |  |  | |
| **Women my age who decide to PLACE THE BABY FOR ADOPTION after becoming pregnant accidentally:** | | | | |
| 1. are usually low income | ☐ Strongly disagree  ☐ Disagree  ☐ Neither agree nor disagree  ☐ Agree  ☐ Strongly agree | 1. usually are not in a committed relationship | ☐ Strongly disagree  ☐ Disagree  ☐ Neither agree nor disagree  ☐ Agree  ☐ Strongly agree | |
| 1. usually do not have family support to raise the child | ☐ Strongly disagree  ☐ Disagree  ☐ Neither agree nor disagree  ☐ Agree  ☐ Strongly agree | 1. are usually well educated | ☐ Strongly disagree  ☐ Disagree  ☐ Neither agree nor disagree  ☐ Agree  ☐ Strongly agree | |

| 1. usually have the child’s best interests at heart | ☐ Strongly disagree  ☐ Disagree  ☐ Neither agree nor disagree  ☐ Agree  ☐ Strongly agree | 1. usually are not ready to be mothers | ☐ Strongly disagree  ☐ Disagree  ☐ Neither agree nor disagree  ☐ Agree  ☐ Strongly agree |
| --- | --- | --- | --- |
| 1. usually have a decent job | ☐ Strongly disagree  ☐ Disagree  ☐ Neither agree nor disagree  ☐ Agree  ☐ Strongly agree | 1. are usually responsible enough to go for checkups during pregnancy | ☐ Strongly disagree  ☐ Disagree  ☐ Neither agree nor disagree  ☐ Agree  ☐ Strongly agree |
| **If you became pregnant accidentally and decided to PLACE THE BABY FOR ADOPTION, how likely is it that the people who matter most to you:** | | | |
| 1. would be disappointed | ☐ Extremely unlikely  ☐ Unlikely  ☐ Neutral  ☐ Likely  ☐ Extremely likely | 1. would be happy | ☐ Extremely unlikely  ☐ Unlikely  ☐ Neutral  ☐ Likely  ☐ Extremely likely |
| 1. would be mad | ☐ Extremely unlikely  ☐ Unlikely  ☐ Neutral  ☐ Likely  ☐ Extremely likely | 1. would be surprised | ☐ Extremely unlikely  ☐ Unlikely  ☐ Neutral  ☐ Likely  ☐ Extremely likely |
| 1. would understand | ☐ Extremely unlikely  ☐ Unlikely  ☐ Neutral  ☐ Likely  ☐ Extremely likely | 1. would not care | ☐ Extremely unlikely  ☐ Unlikely  ☐ Neutral  ☐ Likely  ☐ Extremely likely |
| 1. would be concerned about me | ☐ Extremely unlikely  ☐ Unlikely  ☐ Neutral  ☐ Likely  ☐ Extremely likely | 1. would be supportive | ☐ Extremely unlikely  ☐ Unlikely  ☐ Neutral  ☐ Likely  ☐ Extremely likely |
| 1. would feel ashamed | ☐ Extremely unlikely  ☐ Unlikely  ☐ Neutral  ☐ Likely  ☐ Extremely likely | 1. Adoption is bad for children. | ☐ Strongly disagree  ☐ Disagree  ☐ Neither agree nor disagree  ☐ Agree  ☐ Strongly agree |
| 1. Adoption is difficult for a mother. | ☐ Strongly disagree  ☐ Disagree  ☐ Neither agree nor disagree  ☐ Agree  ☐ Strongly agree | 1. Adoption is better than abortion because the child gets to live. | ☐ Strongly disagree  ☐ Disagree  ☐ Neither agree nor disagree  ☐ Agree  ☐ Strongly agree |
| 1. I understand why some women choose adoption when they get pregnant at a time that they did not plan to. | ☐ Strongly disagree  ☐ Disagree  ☐ Neither agree nor disagree  ☐ Agree  ☐ Strongly agree | 1. If your family will not support you in having a baby, it’s okay to place your child for adoption. | ☐ Strongly disagree  ☐ Disagree  ☐ Neither agree nor disagree  ☐ Agree  ☐ Strongly agree |
| 1. If the man involved in the pregnancy will not support you in having a baby, it’s okay to place your child for adoption. | ☐ Strongly disagree  ☐ Disagree  ☐ Neither agree nor disagree  ☐ Agree  ☐ Strongly agree | 1. If your life is really messed up, it is better to place for adoption than keep the baby. | ☐ Strongly disagree  ☐ Disagree  ☐ Neither agree nor disagree  ☐ Agree  ☐ Strongly agree |
| 1. Women who place the baby for adoption regret it later. | ☐ Strongly disagree  ☐ Disagree  ☐ Neither agree nor disagree  ☐ Agree  ☐ Strongly agree | 1. Imagine that you just found out you became pregnant by accident. How likely would you be to do the following: have a baby and then place it for adoption outside of the family? | ☐ Extremely unlikely  ☐ Unlikely  ☐ Neutral  ☐ Likely  ☐ Extremely likely |
| **Abortion Items** | | | |
| 1. In your opinion, how IRRESPONSIBLE is a woman your age who has an abortion: | ☐ Not at all irresponsible  ☐ A little bit irresponsible  ☐ Somewhat irresponsible  ☐ Quite irresponsible  ☐ Extremely irresponsible | 1. In your opinion, how MATURE is a woman your age who has an abortion: | ☐ Not at all mature  ☐ A little bit mature  ☐ Somewhat mature  ☐ Quite mature  ☐ Extremely mature |
| 1. In your opinion, how SELFISH is a woman your age who has an abortion: | ☐ Not at all selfish  ☐ A little bit selfish  ☐ Somewhat selfish  ☐ Quite selfish  ☐ Extremely selfish | 1. In your opinion, how STRONG is a woman your age who has an abortion: | ☐ Not at all strong  ☐ A little bit strong  ☐ Somewhat strong  ☐ Quite strong  ☐ Extremely strong |
| 1. In your opinion, how COLD/HEARTLESS is a woman your age who has an abortion: | ☐ Not at all cold/heartless  ☐ A little bit cold/heartless  ☐ Somewhat cold/heartless  ☐ Quite cold/heartless  ☐ Extremely cold/heartless |  |  |
| **Women my age who decide to HAVE AN ABORTION after becoming pregnant accidentally:** | | | |
| 1. Are usually financially stable | ☐ Strongly disagree  ☐ Disagree  ☐ Neither agree nor disagree  ☐ Agree  ☐ Strongly agree | 1. Usually are not in a committed relationship | ☐ Strongly disagree  ☐ Disagree  ☐ Neither agree nor disagree  ☐ Agree  ☐ Strongly agree |
| 1. Are usually committed to continuing their education/career | ☐ Strongly disagree  ☐ Disagree  ☐ Neither agree nor disagree  ☐ Agree  ☐ Strongly agree | 1. Usually do not have family support to raise a child | ☐ Strongly disagree  ☐ Disagree  ☐ Neither agree nor disagree  ☐ Agree  ☐ Strongly agree |
| 1. Usually are not well educated | ☐ Strongly disagree  ☐ Disagree  ☐ Neither agree nor disagree  ☐ Agree  ☐ Strongly agree | 1. Usually are not ready to be mothers | ☐ Strongly disagree  ☐ Disagree  ☐ Neither agree nor disagree  ☐ Agree  ☐ Strongly agree |
| 1. Usually have the child’s best interests at heart | ☐ Strongly disagree  ☐ Disagree  ☐ Neither agree nor disagree  ☐ Agree  ☐ Strongly agree | 1. Usually do not have a decent job | ☐ Strongly disagree  ☐ Disagree  ☐ Neither agree nor disagree  ☐ Agree  ☐ Strongly agree |
| **If you became pregnant accidentally and decided to HAVE AN ABORTION, how likely is it that the people who matter most to you:** | | | |
| 1. Would be disappointed | ☐ Extremely unlikely  ☐ Unlikely  ☐ Neutral  ☐ Likely  ☐ Extremely likely | 1. Would be happy | ☐ Extremely unlikely  ☐ Unlikely  ☐ Neutral  ☐ Likely  ☐ Extremely likely |
| 1. Would be mad | ☐ Extremely unlikely  ☐ Unlikely  ☐ Neutral  ☐ Likely  ☐ Extremely likely | 1. Would be surprised | ☐ Extremely unlikely  ☐ Unlikely  ☐ Neutral  ☐ Likely  ☐ Extremely likely |
| 1. Would understand | ☐ Extremely unlikely  ☐ Unlikely  ☐ Neutral  ☐ Likely  ☐ Extremely likely | 1. Would not care | ☐ Extremely unlikely  ☐ Unlikely  ☐ Neutral  ☐ Likely  ☐ Extremely likely |
| 1. Would be concerned about me | ☐ Extremely unlikely  ☐ Unlikely  ☐ Neutral  ☐ Likely  ☐ Extremely likely | 1. Would be supportive | ☐ Extremely unlikely  ☐ Unlikely  ☐ Neutral  ☐ Likely  ☐ Extremely likely |
| 1. Would feel ashamed | ☐ Extremely unlikely  ☐ Unlikely  ☐ Neutral  ☐ Likely  ☐ Extremely likely | 1. If the man involved in the pregnancy will not support you in having a baby, it's okay to have an abortion. | ☐ Strongly disagree  ☐ Disagree  ☐ Neither agree nor disagree  ☐ Agree  ☐ Strongly agree |
| 1. If your life is really messed up, it's better to have an abortion than to keep the baby. | ☐ Strongly disagree  ☐ Disagree  ☐ Neither agree nor disagree  ☐ Agree  ☐ Strongly agree | 1. Abortion is acceptable in any situation. | ☐ Strongly disagree  ☐ Disagree  ☐ Neither agree nor disagree  ☐ Agree  ☐ Strongly agree |
| 1. Abortion should be the woman's decision. | ☐ Strongly disagree  ☐ Disagree  ☐ Neither agree nor disagree  ☐ Agree  ☐ Strongly agree | 1. Abortion is acceptable if the woman cannot take care of a child. | ☐ Strongly disagree  ☐ Disagree  ☐ Neither agree nor disagree  ☐ Agree  ☐ Strongly agree |
| 1. Women should not use abortion as a method of birth control. | ☐ Strongly disagree  ☐ Disagree  ☐ Neither agree nor disagree  ☐ Agree  ☐ Strongly agree | 1. Women who have abortions are killing their own children. | ☐ Strongly disagree  ☐ Disagree  ☐ Neither agree nor disagree  ☐ Agree  ☐ Strongly agree |
| 1. Abortion is the easy way out. | ☐ Strongly disagree  ☐ Disagree  ☐ Neither agree nor disagree  ☐ Agree  ☐ Strongly agree | 1. Abortion is risky for women’s health. | ☐ Strongly disagree  ☐ Disagree  ☐ Neither agree nor disagree  ☐ Agree  ☐ Strongly agree |
| 1. Women who have multiple abortions may not be able to have a child later in life. | ☐ Strongly disagree  ☐ Disagree  ☐ Neither agree nor disagree  ☐ Agree  ☐ Strongly agree | 1. Abortion is acceptable if the woman does not have family support. | ☐ Strongly disagree  ☐ Disagree  ☐ Neither agree nor disagree  ☐ Agree  ☐ Strongly agree |
| 1. Women who have an abortion regret it later. | ☐ Strongly disagree  ☐ Disagree  ☐ Neither agree nor disagree  ☐ Agree  ☐ Strongly agree | 1. Imagine that you just found out you became pregnant by accident. How likely would you be to do the following: have an abortion? | ☐ Extremely unlikely  ☐ Unlikely  ☐ Neutral  ☐ Likely  ☐ Extremely likely |
